# Matrix Metalloproteinases in COPD and atherosclerosis with emphasis on the effects of smoking

**DOI:** 10.1371/journal.pone.0211987

**Published:** 2019-02-21

**Authors:** M. Kraen, S. Frantz, U. Nihlén, G. Engström, C. G. Löfdahl, P. Wollmer, M. Dencker

**Affiliations:** 1 Clinical Physiology and Nuclear Medicine unit, Department of Translational Medicine, Malmö, Lund University, Malmö, Sweden; 2 Respiratory Medicine and Allergology unit, Department of Clinical Sciences, Lund, Lund University, Lund, Sweden; 3 Cardiovascular Epidemiology research group, Department of Clinical Science, Malmö, Lund University, Malmö, Sweden; National and Kapodistrian University of Athens, GREECE

## Abstract

**Background:**

Matrix metalloproteinases (MMP´s) are known biomarkers of atherosclerosis. MMP´s are also involved in the pathophysiological processes underlying chronic obstructive pulmonary disease (COPD). Cigarette smoking plays an important role in both disease states and is also known to affect the concentration and activity of MMP´s systemically. Unfortunately, the epidemiological data concerning the value of MMP´s as biomarkers of COPD and atherosclerosis with special regards to smoking habits are limited.

**Methods:**

450 middle-aged subjects with records of smoking habits and tobacco consumption were examined with comprehensive spirometry, carotid ultrasound examination and biomarker analysis of MMP-1, -3, -7, -10 and -12. Due to missing data 33 subjects were excluded.

**Results:**

The remaining 417 participants were divided into 4 different groups. Group I (n = 157, no plaque and no COPD), group II (n = 136, plaque but no COPD), group III (n = 43, COPD but no plaque) and group IV (n = 81, plaque and COPD). Serum levels of MMP-1,-7,-10-12 were significantly influenced by smoking, and MMP-1, -3, -7 and-12 were elevated in subjects with COPD and carotid plaque. This remained statistically significant for MMP-1 and-12 after adjusting for traditional risk factors.

**Conclusion:**

COPD and concomitant plaque in the carotid artery were associated with elevated levels of MMP-1 and -MMP-12 even when adjusting for risk factors. Further studies are needed to elucidate if these two MMP´s could be useful as biomarkers in a clinical setting. Smoking was associated with increased serum levels of MMP´s (except for MMP-3) and should be taken into account when interpreting serum MMP results.

## Introduction

Matrix metalloproteinases (MMP´s) are a group of structurally related proteins with enzymatic activity collectively involved in the degradation of extracellular matrix (ECM) proteins. They are grouped into collagenases (MMP-1,-8,-13,-14), gelatinases (MMP-2,-9), stromelysins (MMP-3,-10,-11), matrilysins (MMP-7) and macrophage elastase (MMP-12) [1]. Together with their counterpart, tissue inhibitors of metalloproteinases, they are part of an intricate network governing the balance of ECM protein metabolism [1, 2]. In recent years the involvement of MMP´s in the different pathophysiological aspects of chronic obstructive pulmonary disease (COPD) have gained a lot of attention. Especially MMP-9 (but also to a lesser extent MMP-1,-10 and -12) have been implicated in the underlying disease mechanism of obstruction of small airways, development of emphysema, mucus hypersecretion and low grade inflammation assessed at both the local, sputum and broncho-alveolar lavage (BAL), and systemic level [1, 3–7]. But although there is evidence for their involvement at all levels of the disease, the role of MMP´s as serum biomarkers of COPD is relatively unexplored.

MMP´s on the other hand are known serum biomarkers of atherosclerotic disease [8, 9] and are involved in the pathophysiological pathways governing plaque development, stabilization and rupture [10, 11]. It is well known that there is a substantial comorbidity among patients with COPD and atherosclerotic disease [12] and cigarette smoking is arguably the most important common risk factor playing a pivotal role in the pathophysiology of both diseases. Furthermore cigarette smoking is known to affect the concentration and activity of MMP´s both locally and systemically [13–17]. Thus it could be an important confounder which unfortunately is often poorly accounted for. Overall the epidemiological data on MMP´s as biomarkers of COPD in the context of atherosclerosis and smoking are scarce.

Thus the primary aim of the present cross-sectional study was to investigate the potential value of 5 different MMP´s (MMP-1,-3,-7,-10 and -12) as serum biomarkers of COPD and atherosclerosis in a study group with well characterized smoking habits.

## Methods

This was a cross-sectional study performed between 2004–2007 at the Department of Medical Imaging and Physiology, Skåne University Hospital in Malmö. The inclusion procedures and methods involved have previously been described in other publications [18]. The study was approved by the Ethics Committee of Lund University and all participants signed an informed consent form before entering the study.

### Study population

Based on the results of a population questionnaire concerning smoking and lung disease 870 middle-aged subjects (healthy never-smokers, asymptomatic smokers and subjects reporting a diagnosis of COPD) were invited to undergo a comprehensive pulmonary evaluation (spirometry, body plethysmography and diffusing capacity for CO), a carotid ultrasound examination and additional laboratory testing and collection of anthropometric data. 450 subjects accepted the invitation and were enrolled. The recruitment process is described in detail elsewhere [19, 20]. 33 participants were excluded due to missing data on biomarkers (n = 22), diffusing capacity (n = 6) or lipid status (n = 5).

### Lung function tests

Both spirometry and body plethysmography were performed according to ERS recommendations [21] and European reference values were used [21]. A spirometer (Master Screen, Viasys GmbH—Erich Jaeger, Hoechberg, Germany) was used to measure FEV_1_ and vital capacity (VC), while TLC and RV were measured with a body plethysmograph (Master Screen, Viasys GmbH—Erich Jaeger, Hoechberg, Germany). All measurements were performed 15–45 minutes after inhalation of 1.0 mg of terbutaline (Bricanyl^®^ Turbuhaler^®^). Diffusing capacity for carbon monoxide (D_L,CO_) was measured using the single-breath technique [22] (Master Screen, Viasys GmbH—Erich Jaeger, Hoechberg, Germany) and the reference values for D_L,CO_ were corrected for haemoglobin values according to established procedures [23]. The diagnosis of chronic obstructive pulmonary disease (COPD) and severity staging was performed according to recommendations by GOLD (Global initiative for chronic Obstructive Lung Disease) criteria (www.goldcopd.com, 2010).

### Carotid ultrasound examination

The common carotid artery, the bifurcation and the internal carotid artery were examined bilaterally with a linear 7.5 MHz ultrasound probe following standard hospital procedures. The presence (n = 217) or absence (n = 200) of plaque was determined in a semi-quantitatively dichotomous fashion by experienced readers blinded to other study data.

### Smoking habits

Subjects who were currently smoking or had stopped within the last 12 months prior to the study were classified as current smokers. Subjects who stopped smoking more than 12 months prior to the study were classified as ex-smokers. The remaining participants were classified as never-smokers. Total tobacco consumption was calculated in pack years (one pack year = smoking of 20 cigarettes/day for one year). All classifications were based on self-reported smoking habits.

### Blood samples and biomarker analysis

Blood samples were drawn at resting and non-fasting condition. Total cholesterol, high-density lipoprotein (HDL), low density lipoprotein (LDL) and glycated haemoglobin (HbAlc) were measured using routine methods. Plasma EDTA samples were stored in -80°C. MMP-1, -3,-7,-10 and-12 were analyzed by the Proximity Extension Assay technique using the Proseek Multiplex CVD 96x96 reagents kit (Olink Bioscience, Uppsala, Sweden). The coefficients of variance (CoV) of the biomarkers that were analyzed are as follows (intra- and inter-assay variation): MMP-1 (5%, 19%), MMP-3 (9%, 14%), MMP-7 (7%, 11%), MMP-10 (5%, 28%), and MMP-12 (8%, 10%). Data are presented as arbitrary units (AU). Values can be transformed to actual concentrations using transformation algorithms on the Olink Bioscience website (www.olink.com). The conversion, however, is not exact [24, 25].

## Statistics

Statistical analyses were carried out using SPSS Statistics version 24 (IBM, Armonk, NY, USA). Continuous variables are presented as mean ± standard deviation (SD). Categorical variables are presented as numbers or percentages. ANOVA or ANCOVA with Scheffe- or Bonferroni- adjusted p-values were used for group comparison. Correlations were tested with a standard bivariate correlation analysis. Multinomial regression analysis with a standard model and models incorporating MMP´s were performed for predicting group affiliation.

## Results

The final study group consisted of 417 participants who were subsequently stratified according to the presence or absence of COPD or plaque in the carotid artery into 4 different groups. Group I (n = 157, no plaque and no COPD), group II (n = 136, plaque but no COPD), group III (n = 43, COPD but no plaque) and group IV (n = 81, plaque and COPD). Of the 124 subjects who could be diagnosed with COPD the majority were in the mild stages of disease (GOLD I, n = 84, II, n = 35, III, n = 4, IV, n = 1). The clinical characteristics of the total population and the different groups are displayed in Table 1 with all p-values reflecting comparison to group I. As expected the distribution of smoking status was significantly skewed with a preponderance of smokers in group II-IV and very few never-smokers in group III and IV (9% and 4% respectively). Consequently tobacco consumption was also significantly higher in group II-IV. Furthermore, subjects in group II-IV were older and with a male preponderance. In line with the smoking data D_L,CO_ was reduced and residual volume significantly elevated in groups III-IV. Total lung capacity (TLC) was significantly raised in group III only. Systolic blood pressure was higher in subjects with carotid plaques regardless of concomitant COPD. There were no differences groups for lipids and HbA1c. Regarding MMP´s we found that all serum MMP levels except MMP-10 were significantly elevated in group IV. MMP-7 and-12 were also elevated in subjects with plaque, whereas MMP-1 was raised in group II-IV. Correlation analysis (Table 2) showed a positive correlation with tobacco consumption and all MMP´s, and with age for MMP-3,-7 and -12. As shown in Table 3 the levels of MMP´s were significantly influenced by smoking status especially by current smoking and primarily for MMP-1,-10 and-12.

**Table 1:** Data on anthropometrics, pulmonary, clinical and biochemical variables. Group I (no plaque or COPD), group II (plaque without COPD), group III (COPD without plaque), group IV (plaque and COPD). Values are mean ±SD or numbers. ANOVA or Chi ^2^ were used for calculating p-values, which reflects comparison to group I. Scheffe was used as post hoc test for multiple comparisons.

Group Variable	I (n = 157)	II (n = 136)	III (n = 43)	IV (n = 81)	Total (n = 417)
Sex (male/female)	47/110	63/73*	19/24	45/36**	174/243
Age (years)	58 ±7.3	63 ±7.1***	63 ±7.1***	66 ±6.2***	62 ±7.6
BMI (kg/m^2^)	27 ±5.3	27 ±5.2	26 ±4.5	26 ±4.6	27 ±5.1
Smoking habits (n/ex/cu)	48/49/60	24/37/75**	4/19/20	3/28/50***	79/133/205
Pack years (years)	14 ±14	24 ±18***	23 ±16*	35 ±23***	23 ±19
Systolic BP (mmHg)	134 ±17	141 ±18*	137 ±20	143 ±20**	138 ±18
HbA1c (%)	4.6 ±0.4	4.8 ±0.8	4.7 ±0.5	4.9 ±0.9*	4.7 ±0.7
Cholesterol (mmol/L)	5.8 ±1.0	5.7 ±1.1	6.1 ±0.8	5.5 ±1.1	5.8 ±1.0
HDL (mmol/L)	1.4 ±0.4	1.3 ±0.4	1.4 ±0.4	1.2 ±0.4	1.3 ±0.4
LDL (mmol/L)	3.8 ±0.9	3.8 ±1.0	4.0 ±0.8	3.6 ±1.1	3.8 ±1.0
FEV_1_ (%pred)	107 ±14	103±15	91±17***	83±17***	99±18
VC (%pred)	112 ±14	108 ±15	116 ±16	108 ±17	110±16
FEV_1_/VC (%pred)	102 ±6	101 ±6	83 ±9***	81 ±11***	96 ±12
RV (%pred)	100 ±17	105 ±20	123 ±21***	118 ±26***	108 ±22
TLC (%pred)	102 ±11	102 ±11	112 ±12***	105 ±13	104 ±12
RV/ TLC (%pred)	94 ±11	97 ±14	103 ±17**	105 ±17***	98 ±14
D_L,CO_ (%pred)	87 ±14	83 ± 16	81 ± 21	70 ±15***	82 ±17
MMP-1 (AU)	1.25 ±0.86	1.46 ±0.95	1.57 ±0.94	1.71 ±0.85 **	1.44 ±0.91
MMP-3 (AU)	1.85 ±0.79	1.96 ±0.73	1.98 ±0.94	2.23 ±0.84 **	1.97 ±0.81
MMP-7 (AU)	5.85 ±0.61	6.09 ±0.71*	5.85 ±0.51	6.17 ±0.74 **	5.99 ±0.67
MMP-10 (AU)	7.54 ±0.70	7.59 ±0.69	7.49 ±0.56	7.53 ±0.77	7.55 ±0.70
MMP-12 (AU)	6.46 ±0.70	6.90 ±0.75 ***	6.55 ±0.77	7.20 ±0.89 ***	6.76 ±0.81

Abbreviations: Body mass index (BMI), never smokers (n), ex-smokers (ex), current smokers (cu), blood pressure (BP). Diffusing capacity (D_L,CO_), vital capacity (VC), forced expiratory volume (FEV_1_), residual volume (RV) and total lung capacity (TLC) all in percent of predicted (%pred). Arbitrary units (AU).

* Indicates significant difference (P<0.05) compared to group I.

** Indicates significant difference (P<0.01) compared to group I.

*** Indicates significant difference (P<0.001) compared to group I.

**Table 2:** Correlations analysis (Pearson`s r) between MMP`s and clinical and pulmonary variables.

	MMP-1	MMP-3	MMP-7	MMP-10	MMP-12
Age	0.06	0.30***	0.25***	0.04	0.35***
Pack years	0.11*	0.19***	0.13**	0.12*	0.31***
Systolic BP	0.00	0.06	0.09	-0.14**	0.09
HbA1c	0.09	0.11*	0.12*	0.09	0.23***
LDL	-0.03	-0.11*	-0.07	-0.11*	-0.10*
FEV_1_ (%Pred)	-0.20***	-0.04	-0.11*	-0.01	-0.25***
VC (%Pred)	-.009	-0.06	-0.04	-0.02	-0.16**
FEV_1_/VC (%Pred)	-0.19***	-0.11*	-0.10*	0.01	-0.18***
RV (%Pred)	0.02	-0.06	0.02	0.01	0.08
TLC (%Pred)	-0.04	-0.10*	-0.04	-0.03	-0.08
RV/TLC (%Pred)	0.06	-0.11*	0.07	0.01	0.17***
D_L,co_ (%Pred)	-0.24***	0.03	-0.22***	-0.13*	-0.37***

Abbreviations: Blood pressure (BP). Diffusing capacity (D_L,CO_), vital capacity (VC), forced expiratory volume (FEV_1_), residual volume (RV) and total lung capacity (TLC) all in percent of predicted (%pred). Low density lipoprotein (LDL), glycosylated haemoglobin (HbA1c)

* Indicates (p<0.05)

** Indicates (p<0.01)

*** Indicates (p<0.001)

**Table 3:** ANCOVA analysis of MMP values stratified by smoking status with age-adjusted p-values. Bonferroni was used as post hoc test due to multiple comparisons. Values are mean ± SD.

	Never-smokers (n = 79)	Ex-smokers (n = 133)	Current smokers (n = 205)
MMP-1	1.16 ±0.96	1.31 ±0.88^¤¤^	1.64 ±0.86***
MMP-3	1.89 ±0.82	2.11 ±0.90	1.92 ±0.72
MMP-7	5.83 ±0.56	5.98 ±0.69	6.06 ±0.67**
MMP-10	7.30 ±0.60	7.46 ±0.73^¤¤^	7.69 ±0.68***
MMP-12	6.40 ±0.68	6.62 ±0.81^¤¤¤^	6.98 ±0.79***

* Indicates significant difference (P<0.05), compared to never-smokers.

** Indicates significant difference (P<0.01), compared to never-smokers.

*** Indicates significant difference (P<0.001), compared to never-smokers.

¤ Indicates significant difference (P<0.05), compared to current smokers.

¤¤ Indicates significant difference (P<0.01), compared to current smokers.

¤¤¤ Indicates significant difference (P<0.001), compared to current smokers

Based on the findings mentioned above and to test whether MMP´s possessed predictive capacity of group affiliation, we performed a multinomial regression analysis using a standard model consisting of age, gender, smoking status, pack years, blood pressure, HbA1c and LDL with subsequent addition of the MMP´s individually. The results are displayed in Table 4 which shows that in the standard model age, blood pressure, pack years and smoking status were significant predictors with the highest odds ratio for smoking status. Only MMP-1 and MMP-12 contributed significantly to this model with odds ratios of 1.64 and 1.60 respectively.

**Table 4:** Multinomial regression analysis with group comparison. Group I (n = 157, no plaque and no COPD), group II (n = 136, plaque but no COPD), group III (n = 43, COPD but no plaque) and group IV (n = 81, plaque and COPD). MMPs were added individually to the standard model that included sex, age, smoking status, pack years, systolic BP, HbA1c and LDL.

Group	II vs I OR (95% CI)	III vs I OR (95% CI)	IV vs I OR (95% CI)
Male sex	1.34 (0.78–2.33)	1.40 (0.73–3.42)	1.40 (0.63–2.53)
Age (per year)	1.10 (1.06–1.14)***	1.12 (1.06–1.18)***	1.17 (1.11–1.23)***
Exsmokers vs neversmokers	0.87 (0.36–2.12)	3.33 (0.91–16.66)	3.44 (0.79–15.03)
Current vs neversmokers	1.73 (0.68–4.41)	3.81 (0.96–14.85)	6.78 (1.39–28.38)*
Pack years (per year)	1.04 (1.02–1.07)***	1.03 (1.00–1.06)	1.06 (1.03–1.09)***
Systolic BP (per mmHg)	1.03 (1.01–1.04)**	1.01 (1.00–1.03)	1.03 (1.01–1.05)**
HbA1c (per %)	1.00 (0.67–1.65)	0.85 (0.45–1.71)	1.22 (0.72–1.91)
LDL (per mmol/L)	0.96 (0.73–1.26)	1.34 (0.88–1.87)	0.79 (0.57–1.13)
MMP-1 (per unit)	1.26 (0.94–1.70)	1.48 (0.99–2.22)	1.64 (1.13–2.36)**
MMP-3 (per unit)	0.70 (0.46–1.02)	0.61 (0.42–1.22)	0.95 (0.55–1.44)
MMP-7 (per unit)	1.34 (0.86–2.01)	0.69 (0.41–1.37)	1.27 (0.76–2.06)
MMP-10 (per unit)	0.90 (0.61–1.30)	0.69 (0.44–1.28)	0.70 (0.40–1.08)
MMP-12 (per unit)	1.37 (0.93–2.02)	0.79 (0.46–1.32)	1.60 (1.01–2.56)*

* Indicates significant difference (P<0.05)

** Indicates significant difference (P<0.01)

*** Indicates significant difference (P<0.001)

## Discussion

In the present cross sectional study we explored the associations between MMP´s (1, 3,7,10 and 12) and carotid plaque and COPD with special regards to smoking status and tobacco consumption. Our main finding was that serum levels of MMP-1 and -12 in a multivariate regression model were independent predictors of concomitant COPD and carotid plaque with odds ratios of 1.6 and 1.5 respectively. This means that in our study population MMP-1 and -12 levels in itself carry information about clinical pulmonary disease and subclinical vascular disease even when accounting for traditional risk factors especially smoking habits. Admittedly, background data on relevant comorbidity which could be biasing the results are lacking, and the numerical differences are small and with relatively large confidence intervals which probably prohibits the use of MMP´s as biomarkers in a clinical setting. But we nevertheless consider this an important epidemiological finding.

Not surprisingly the MMP´s differed somewhat in their profiles regarding their association with plaque, COPD and smoking: MMP-1 was significantly associated with both COPD and plaque and the combination hereof even in multivariable analysis. MMP-3 was only elevated in group IV and this was probably an age-related finding. MMP-7 seemed primarily a marker of atherosclerosis, but was influenced by smoking and age. MMP-10 was influenced by smoking habits but was apart from that generally unaffected by COPD or plaque status. Finally MMP-12 seemed primarily a marker of atherosclerosis, but was also significantly associated with the combination of COPD and plaque even when adjusting for several risk factors. Concerning MMP´s and their association with atherosclerosis our findings are well in line with the results of a previous larger study by Goncalves et al [8]. In this study (in a subgroup of non-diabetics, n = 515) it was shown that serum levels of MMP-7 and -12 were significantly elevated in subjects with signs of cardiovascular disease (n = 270) whereas levels of MMP-1,-3 and-10 were unaffected.

Another important finding in our study is the strong influence that current smoking and tobacco consumption exerts on the levels of MMP-1,-7,-10 and-12. This is in line with the results of previous studies. In a recent large population based study of the effects of smoking on a wide variety of biomarkers, MMP-1,-10 and -12 were significantly affected by current smoking and these findings could be replicated in another subsequent cohort [16]. In this study lung function testing was not performed. In a study (74 COPD subjects and 20 controls) serum levels of MMP-1, -3 and -7 were found to be significantly increased in COPD and MMP-1 was also increased in smokers [26]. The COPD subjects were mainly in GOLD stage II and the MMP measurements were done with a different technique of microsphere analysis. With the aid of induced sputum analysis Culpitt et al. showed levels of MMP-1 (but not MMP-3) to be increased in smokers and subjects with more advanced COPD [27]. This was a small study with 15 subjects in each group. Finally, in a study of 53 COPD subjects and 46 controls the sputum levels of MMP-12 was found to be elevated in COPD subjects and in healthy smokers as compared to healthy non-smokers [13]. Taken all together the findings from these studies suggest associations between MMP-1 and-12 and COPD although they are relatively small and performed in patients with more advanced disease. Moreover, they are in general lacking detailed analysis of the impact of smoking and coexisting atherosclerosis.

MMP-1, which degrades collagen, and MMP-12, which degrades elastin, have both been strongly implicated in the development of smoke-induced emphysema, at least in animal models [15, 28, 29]. Furthermore numerous experimental studies have shown MMP-1 and -12 to be implicated in plaques development [30–33]. But unfortunately sound epidemiological data on serum MMP´s in the setting of COPD and concomitant plaque are scarce and there is a giant gap from experimental studies to clinical epidemiology. So in this perspective we believe that the main findings of our study could be an important step in trying to bridge that gap.

## Limitations

The main limitation of this study is the cross-sectional study design prohibiting prospective analysis of prediction and prognosis. The study is population based, but due to recruitment procedures the study group cannot fully represent the general population. Smoking status and tobacco consumption was based on subjective self-reports which of course introduces some uncertainty. In our study the presence of carotid plaque was used as a surrogate marker of general atherosclerosis, which of course is questionable, but we consider this justifiable when used only for stratification purposes. Also data on relevant comorbidity such as diabetes and cardiovascular disease are lacking and could therefore not be incorporated in the regression model. MMP-9 is arguably the most studied MMP with regards to COPD but unfortunately this was not a part of the proteomic analysis kit and hence data on this specific MMP was not available to us. Odds ratios for MMP´s in the multinomial regression analysis should be interpreted with caution as the levels of MMP´s are expressed in arbitrary units. Finally, it has to be considered that a single serum measurement of a protein involved in complex pathophysiological pathways at cellular level is a very crude estimate of subclinical or clinical disease.

## Conclusion

Serum levels of MMP-1,-7, -10 and-12 are influenced by current smoking and MMP-1, -3, -7 and-12 are elevated in subjects with COPD and carotid plaque at the early stages of disease. These associations remain significant for MMP-1 and-12 after adjusting for traditional risk factors and smoking habits. Albeit the numerical differences are small so further studies are needed to elucidate if MMP´s could be used as biomarkers in a wider clinical setting.
